# Association between Rheumatoid Arthritis and Respiratory Allergic Diseases in Korean Adults: A Propensity Score Matched Case-Control Study

**DOI:** 10.1155/2018/3798124

**Published:** 2018-04-23

**Authors:** Han Eol Jeong, Sung-Mok Jung, Sung-Il Cho

**Affiliations:** ^1^Department of Public Health Science, Graduate School of Public Health, Seoul National University, Seoul, Republic of Korea; ^2^Graduate School of Medicine, Hokkaido University, Sapporo, Japan; ^3^Institute of Health and Environment, Seoul National University, Seoul, Republic of Korea

## Abstract

Rheumatoid arthritis (RA) and allergic diseases are result of a poor functioning immune system, giving dominance to either T-helper 1 (Th1) or T-helper 2 (Th2) diseases, respectively. Studies have stated that there seems to be a relationship present between the immune response subsets. This study was designed to examine the association between RA and respiratory allergic diseases in Korean adults. The study utilized the KNHANES 2013–2015 data and excluded individuals diagnosed with RA before being diagnosed with allergic diseases, using age at clinical diagnosis. Total of 253 RA patients were matched 1 : 1 with non-RA patients by a propensity score, using sex and age as matched variables. Multivariate conditional logistic regression analyses were used to evaluate for association between RA and respiratory allergic diseases in the matched 506 participants. RA was associated with an increased risk of prevalence of respiratory allergic diseases with an OR of 1.51 (95% CI, 1.31–1.75), adjusted for socioeconomic demographic variables. The adjusted OR for prevalence of RA among participants with prevalence of asthma and allergic rhinitis was as follows: 3.12 (95% CI, 2.77–3.51) and 1.39 (95% CI, 1.16–1.67). Participants with prevalence of asthma in particular had an increased risk of developing prevalence of RA. Based on our findings, Th1 and Th2 diseases may indeed coexist, and one pathway may stimulate or contribute towards the onset of the other.

## 1. Introduction

There are two main immune pathways present within our body that act as a line of defense when foreign pathogens enter the body. The key factor differentiating the two immune pathways, T-helper 1 (Th1) and T-helper 2 (Th2), is the distinctive cytokine pattern expression [1]. T-helper cells are a subtype of white blood cells called lymphocytes that detect and recognize foreign pathogens or, in the case of autoimmune diseases, normal body tissues. Cytokines are hormonal messenger proteins that are responsible for the biological effects of the immune system [1]. Some examples of Th1 dominant diseases are type I diabetes mellitus, multiple sclerosis, rheumatoid arthritis (RA), coeliac disease, and its counterpart; Th2 dominant diseases are lupus, allergic rhinitis, inflammatory bowel disease, and asthma [1–3].

Immune response involving Th1 cells has the tendency to produce interferon-gamma (IFN-gamma) and interleukin-2 (IL-2) cytokines and participate in delayed-type hypersensitivity (DTH) and macrophage activation [4]. On the contrary, immune response involving Th2 cells produce interleukin-4 (IL-4), interleukin-5 (IL-5), interleukin-10 (IL-10), and interleukin (IL-13) cytokines and are involved in allergic and its relevant responses to parasites [4]. A downside to the Th1 immune response is that it is often considered to be the more destructive response of the two; hence, when overreactive, it may lead to organ-specific autoimmune diseases, in this case, RA in the joints [5], whereas a negative side of the Th2 pathway is that it is regarded to be an underlying allergy and related immunoglobulin E- (IgE-) based disease and predisposing to systemic autoimmune diseases, for instance, allergic diseases such as allergic rhinitis and asthma.

Allergic rhinitis and asthma are few of the well-known Th2 autoimmune diseases. They are known to be of a genetically predetermined disorder, where the IgE antibody is created in response to specific allergens [6]. According to a 13 years' worth of data released by the Korea Centers for Disease Control and Prevention (KCDC), the number of patients with allergic diseases has increased more or less in a stable rate as time passed. Allergic rhinitis patients increased the most over 13 years, from 1.2% in 1998 to 14.5% in 2011, which is 13.3% increase. Asthma patients also increased on the whole but at a smaller quantity, from 1.2% in 1998 to 3.0% in 2011.

On the other hand, RA, as mentioned above, is a Th1 autoimmune disease, arising due to the inflammatory reaction of the immune system against self-body tissues, eventually leading to physical disability by the destruction of joints [6, 7]. According to the National Health Insurance System (NHIS), over the past five years from 2010 to 2014, the number of RA patients has increased at a steady rate from 73,215 patients in 2010 to 94,601 in 2014. This is 29% increase in the number of RA patients in just five years.

Both RA and allergic diseases are result of a poor functioning immune system, giving dominance to either Th1 or Th2 diseases, respectively. However, in the case of a well-functioning, healthy immune system, both Th1 and Th2 pathways work without disability and in harmony to maintain and regulate the body's immune system in check and balance. One of the two pathways could become more activated in response to a foreign pathogen but would revert back to its stable level once that threat has been eradicated.

Number of studies done previously have stated that there seems to be a relationship, whether positive or negative, present between the two immune response subsets, taking into consideration that the two subsets, Th1 and Th2, produce cytokines that cross-regulate each counterpart's growth and activity [1]. Therefore, in this study, we attempted to determine the relationship between RA and respiratory allergic diseases and the underlying relationship of Th1 and Th2 diseases through a cross-sectional population-based study done in South Korea. To the best of our knowledge, this is the first nationwide population-based study examining the relationship between respiratory allergic disease and its risk of RA.

## 2. Methods

### 2.1. Data

This study utilized data obtained from cross-sectional Korea National Health and Nutrition Examination (KNHANES) from 2013 to 2015. KNHANES was and is still conducted annually by the KCDC and the Ministry of Health and Welfare (MoHW) of South Korea. A stratified multistage clustered probability sample design was used to generate the representative data among the noninstitutionalized Korean population. All statistics of KNHANES have been calculated using sample weights assigned to sample participants (sample weights were constructed for sample participants to represent the Korean population by accounting for the complex survey design, survey nonresponse, and poststratification). The weights based on the inverse of selection probabilities and inverse of response rates were modified by adjusting them to the sex and age of specific Korean populations, or also known as poststratification [8].

### 2.2. Study Subjects

All subjects under the age of 19 were excluded. Among the total number of subjects that participated in the KNHANES, subjects who did not respond to any of the following questions: “Have you ever been diagnosed as having asthma by a physician” and “Have you ever been diagnosed as having allergic rhinitis by a physician” were excluded, while subjects that responded “yes” and subjects that responded with a “no” were included in the study [8]. As a result, 42 subjects were defined as subjects with allergic disease. The aforementioned process was undergone for RA in the same manner, in this case, the question being, “Have you ever been diagnosed as having RA by a physician.” Moreover, KNHANES data includes not only the presence or absence of disease, but also the age at clinical diagnosis of each disease. Therefore, using the age data of clinical diagnosis, only those diagnosed with allergic diseases before being diagnosed with RA were included in the study, whereas, the counterparts, those diagnosed with allergic diseases at an older age than the age of RA diagnosis, were excluded. The minimum interval required for the age of diagnosis of respiratory allergic diseases and that of RA were defined as one year old. This was done to solely examine the relationship between RA and respiratory allergic diseases among those already diagnosed with respiratory allergic diseases prior to being diagnosed with RA, allowing temporal information to be reviewed within a propensity score matched case-control study that utilized cross-sectional data.

### 2.3. Covariates

Clinical characteristic data were collected by history taking and physical examinations. Basic demographics, such as sex, age, marital status, household income, cigarette smoking and alcohol drinking, and prevalence of diabetes mellitus (normal, impaired fasting glucose, and diabetes mellitus) were included in the history taking. As for physical examinations, weight and height to calculate the body mass index (BMI) and waist circumference (WC) were included. Weight was measured using a calibrated balance-beam scale and height was measured using a stadiometer at an upright position. BMI was then calculated using the measured values of weight and height by dividing weight (kg) by (height)^2^ (m^2^). WC was measured at the midpoints between the bottom of the ribcage and at the top of the lateral border of the iliac crest at full expiration.

### 2.4. Statistical Analysis

All statistical analyses were conducted using SAS (SAS version 9.4; SAS Institute, Cary, NC, USA). In order to take into account the noninstitutionalized Korean population's representative estimates, survey sample weights, which were calculated using the sampling rate, response rate, and age to sex proportions of the reference population at that specific survey year's Korean National Census registry, were applied to all analyses. Based on the outcome, prevalence of RA, the case (RA group) and control (non-RA group) were matched by propensity score (PS) on a ratio of one to one, respectively, for sex and age. Rao-Scott chi-square test, using PROC SURVEYFREQ of SAS, was used for baseline descriptive statistics for all variables, and a *p* value of less than 0.05 was considered to be statistically significant. Multivariable conditional logistic regression analysis, using PROC SURVERYLOGISTIC of SAS, with PS matching and taking the weights into consideration, was adjusted for the following: socioeconomic and lifestyle characteristics (sex, age, household income, cigarette smoking, alcohol drinking, occupation, obesity, BMI, and diabetes mellitus). In order to determine the association between RA and respiratory allergic diseases for each of the aforementioned variables, both crude and adjusted odds ratios (OR) with 95% confidence intervals (CI) were calculated and a *p* value of less than 0.05 was considered to be statistically significant.

### 2.5. Ethics

KNHANES 2013–2015 was performed by the MoHW and the KCDC of South Korea. All participants in the survey provided written informed consent. KNHANES was approved by the Institutional Review Board (IRB) of Seoul National University, South Korea (IRB no. E1711/003-017), and this study was conducted according to the Declaration of Helsinki in 2013.

## 3. Results

From the KNHANES cross-sectional study, a total of 506 participants met the inclusion criteria. Among the included total study participants, following the results of the PS match as aforesaid, 253 participants were classified into the non-RA or control group and 253 participants were classified into the RA or case group. As the control and case groups were matched by PS on sex and age, the two group's Rao-Scott chi-square test's *p* value for sex and age was 0.8582 and 0.7652, respectively, inferring that the PS match was successful as intended and that there are no significant differences between the two groups with respect to sex and age. As for prevalence of respiratory allergic diseases, there was a difference between the control and case group, with 29 participants having prevalence in both RA and respiratory allergic diseases. For other socioeconomic demographic variables, only obesity had a significant difference between the two groups, whereas the remaining variables had no significant differences (Table 1).

The adjusted odds ratio (OR^A^) for participants with prevalence of RA in participants with prevalence of respiratory allergic diseases (asthma and allergic rhinitis) is shown in Table 2. On the whole, after controlling and adjusting for potential confounders: sex, age, household income, marital status, cigarette smoking, alcohol drinking, occupation, obesity, BMI, and diabetes mellitus, participants with a prevalence of respiratory allergic diseases had a significantly higher risk of prevalence of RA, with an OR^A^ of 1.52 (95% CI, 1.31–1.75) and the OR^A^ was statistically significant as the *p* value was less than 0.05. For those who are current smokers, they had a statistically significant higher risk of developing prevalence of RA, when compared with never or former smokers, with an OR^A^ of 1.51 (95% CI, 1.24–1.85). Moreover, females and married participants had a statistically significant higher risk of developing prevalence of RA compared to that of males and single participants, with an OR^A^ of 1.42 (95% CI, 1.11–1.83) and 1.72 (95% CI, 1.46–2.01), respectively.

The stratified crude OR (OR^C^) and OR^A^ for participants with prevalence of RA in participants with prevalence of either of the two different types of respiratory allergic diseases (asthma and allergic rhinitis) are shown as both grouped and on its own, independently in Table 3. In general, viewing the respiratory allergic diseases separately, participants with a prevalence of asthma had the greatest risk of prevalence of RA, with a OR^C^ of 2.81 (95% CI, 2.60–3.05). The second highest was participants with prevalence of allergic rhinitis with a OR^C^ of 1.37 (95% CI, 0.98–1.90), but this OR^C^ was statistically insignificant as the *p* value was greater than 0.05. Upon controlling and adjusting for potential confounders: sex, age, household income, cigarette smoking, alcohol drinking, occupation, obesity, BMI, and diabetes mellitus, equivalent order of risk was still observed with prevalence of asthma being the highest risk of prevalence of RA with an OR^A^ of 3.12 (95% CI, 2.77–3.51), with allergic rhinitis following behind with an OR^A^ of 1.39 (95% CI, 1.16–1.67). Except OR^C^ of allergic rhinitis and allergic rhinitis without asthma and OR^A^ of allergic rhinitis without asthma, the remaining was all statistically significant with a *p* value of less than 0.05. Furthermore, by excluding participants with concurrent prevalence of both asthma and allergic rhinitis, and also adjusting for the potential confounders as mentioned above, the OR^A^ for prevalence of allergic rhinitis without asthma was 1.03 (95% CI, 0.83–1.28) and prevalence of asthma without allergic rhinitis was 1.92 (95% CI, 1.72–2.14).

## 4. Discussion

Through our cross-sectional study using KNHANES data from 2013 to 2015, it was clearly found that participants with prevalence of respiratory allergic diseases (asthma and allergic rhinitis) had a significantly increased risk of prevalence of RA. This claim is valid with reasonable certainty as of those diagnosed with RA; only participants diagnosed at an older age than the age of respiratory allergic disease diagnosis were included in the study. In short, all RA cases included in the study were diagnosed with RA after they have been diagnosed with respiratory allergic diseases at a younger age. Despite being a cross-sectional study, this allowed for a clearer temporal relationship to be seen for the risk of RA in those with prevalence of respiratory allergic diseases. Among the participants with prevalence of respiratory allergic diseases, participants with prevalence of asthma, in particular, had a higher risk compared to that of allergic rhinitis. According to a nationwide population-based cohort study done in Taiwan [9], they found parallel results, where patients with allergic diseases, in particular, asthma and allergic rhinitis, had a significantly increased risk of developing RA. Likewise with our study results, patients with asthma exhibited the highest risk of developing RA (adjusted hazards ratio (AHR) 1.70 [95% CI, 1.32–2.10]), with patients with allergic rhinitis (AHR 1.62 [95% CI, 1.33–1.98]) and atopic dermatitis (AHR 1.41 [95% CI, 0.98–2.02]) following behind. Moreover, a study based on the Swedish Hospital Discharge Register stated that asthmatic patients had an increased risk of RA [10]. However, a contrasting result, where a cross-sectional study was done in Israel using the Israeli Defense Force database, reported that compared with asthmatic people, nonasthmatic people had a higher prevalence of RA (risk ratio (RR) 2.17 [95% CI, 1.37–3.43]) [11]. They concluded that asthma status may affect the prevalence of RA and preexisting asthma seemed to protect against the development of RA and other autoimmune diseases, rather than increasing the risk.

There exist two opposing views on the underlying relationship between Th1 (RA) and Th2 (respiratory allergic diseases) diseases, with one being a positive association [12, 13] and the other being a negative or inverse association [14, 15]. With regard to positive association, a Finnish cohort study has indicated that the cumulative incidence of asthma in children with coeliac disease or RA was significantly higher than in children without coeliac disease or RA, concluding that Th1 and Th2 diseases could indeed coexist [16]. Furthermore, a review on the relationship between asthma and RA [17] and a study between asthma and proinflammatory conditions [18], done by the Mayo Medical School of Rochester, USA, pointed out that although a population-based cohort study is required utilizing a well-defined population, the standing evidence from these studies found through the review is quite convincing and strongly supports the positive association between asthma and the risk of RA. Conversely, a study done in Germany stated an opposing result supporting that there exists an inverse association between Th1 and Th2 diseases, where RA and atopy antagonize each other and that a change in the cytokine patterns of Th1 and Th2 cells could provide an indication for curative effect on RA [19].

Several previous studies have suggested few potential mechanisms underlying the positive association present between respiratory allergic diseases and the increased risk of prevalence of RA [17]. Possible mechanisms are genetic, environmental factors, natural killer group 2D (NKG2D), and tumor necrosis factor alpha (TNF-alpha). As for genetics, HLA-DRB1 and HLA-DQB1 genes were independently associated with asthma and its related traits in numerous candidate gene association studies, and HLA-DRB1 was found to be the major determinant of the association with RA susceptibility [20]. Similar to genetics, a gene-environmental interaction could be a potentially key pathway accounting for the association between asthma and RA, as studies have proposed that smokers, an environmental factor, are at increased risk of developing asthma [21] and RA [22], independently. Various studies claimed that, amongst a subset of asthmatics, increased NKG2D activity in immune cells may contribute towards initiation of autoimmunity that accelerates development of RA [23–25]. Lastly, activated TNF-alpha pathway in patients with severe asthma may continue to worsen T-regulatory (T-reg) function, which then results in an imbalance between T-reg cells and pathogenic Th17 and Th1 cells in the synovial cells, eventually leading to the development of RA [26, 27].

To the best of our knowledge, this study is the first to show that people with respiratory allergic diseases have an increased risk of prevalence of RA in South Korea, with the two major types of respiratory allergic diseases (asthma and allergic rhinitis) and its inner-relationship among these two taken into consideration. As currently, a clear mechanism with hard scientific evidence behind either RA (Th1) or respiratory allergic diseases (Th2) is unknown; further studies and research in this field are critical in resolving the principal relationship between these two immune responses. Moreover, through our findings, as there seems to be some form of connection amongst the respiratory allergic diseases, in contributing to the risk of prevalence of RA, a common pathogenic mechanism may be shared by the allergic diseases.

Strengths of our study are that although there have been studies done in the past where they have examined the relationship between RA and allergic diseases [2, 3, 6, 28, 29], these studies have either a very limited number of subjects or are confined to a small regional area. Hence, in order to overcome this, we utilized a nationwide surveillance database that well represents the entire South Korean population rather than focusing on a specific region of interest. Likewise, unlike other previous studies, which focused on the relationship between asthma alone, we examined the relationship between two respiratory allergic diseases, allergic rhinitis on top of asthma, with regard to its risk of prevalence of RA. In addition, to appreciate the underlying temporal relationship that may exist, only RA cases that were diagnosed at an age older than the respiratory allergic diseases diagnosis age were included in the study. This allowed for a limited temporal relationship to be observed between respiratory allergic diseases and RA. Hence, through our study, it was clearly shown that there exists a positive association between respiratory allergic diseases and RA and that Th1 and Th2 diseases may indeed coexist and not antagonize one another.

Nevertheless, our study has several limitations when it comes to interpreting and applying the results of this study. First, KNHANES, the data used in this study, is a cross-sectional survey, giving rise to the absence of clear and definite temporal relationship between the exposure and outcome under study. Second, as RA can be difficult to diagnose in its early stages due to similar symptoms with other diseases and the lack of a definitive test for the conditions, the gap between the real incidence date of RA and the physician-confirmed RA diagnosis date could have occurred. In addition, only a relatively small number of participants with respiratory allergic diseases and RA were included in the study which could have caused a minor influence on the high OR values. Despite all the mentioned above, our study's findings support the hypothesis that Th1 and Th2 diseases could coexist and that prevalence of respiratory allergic diseases increases the risk of RA.

## 5. Conclusions

In conclusion, participants with prevalence of respiratory allergic diseases, in particular asthma, had an increased risk of prevalence of RA. Based on our findings, Th1 and Th2 diseases may indeed exist simultaneously, and one pathway could stimulate or contribute towards the onset of the other. Hence, prevalence of respiratory allergic diseases should be considered for use as a potential predicting factor of prevalence of RA and people with prevalence of respiratory allergic diseases should be advised to behave in ways to prevent the development of RA.

## Figures and Tables

**Table 1:** Baseline characteristics of the study population in KNHANES 2013–2015.

	Non-RA (*N* = 253)			RA (*N* = 253)			*p* value
	*N*	Weighted *N*	Weighted %, (95% CI)	*N*	Weighted *N*	Weighted %, (95% CI)	
Prevalence of respiratory allergic diseases							
No respiratory allergic diseases	230	665765	91.2 (87.1–95.3)	224	651567	86.3 (80.9–91.7)	<0.0001
Respiratory allergic diseases	23	64381	8.8 (4.7–12.9)	29	103676	13.7 (8.3–19.1)	
Sex							
Male	58	204863	28.1 (21.7–34.4)	58	214759	28.4 (21.6–35.3)	0.8582
Female	195	525284	71.9 (65.6–78.3)	195	540484	71.6 (64.7–78.4)	
Age (years)							
<41	17	71815	9.8 (5.2–14.5)	17	78921	10.4 (5.2–15.7)	0.7652
41–59	86	298912	40.9 (33.9–48.0)	86	318631	42.2 (34.7–49.6)	
59<	150	359419	49.2 (42.1–56.3)	150	357690	47.4 (39.9–54.8)	
Education level							
Elementary school or lower	113	279860	38.3 (31.4–45.2)	124	318538	42.2 (35.4–48.9)	0.0324
Middle school	38	109028	14.9 (9.5–20.3)	38	105149	13.9 (9.2–18.6)	
High school	51	199620	27.3 (20.7–34.0)	51	181359	24 (17.6–30.5)	
College or higher	41	141638	19.4 (13.7–25.1)	40	150197	19.9 (13.8–25.9)	
Household income (1,000 KRW)							
<200	120	303271	41.5 (34.5–48.5)	129	350605	46.4 (39.8–53.1)	<0.0001
200–450	90	294685	40.4 (33.2–47.6)	64	191559	25.4 (18.9–31.9)	
450<	43	132190	18.1 (12.8–23.4)	60	213079	28.2 (21.6–34.9)	
Marital status							
Single	190	554807	76.0 (69.8–82.2)	171	507101	67.1 (60.2–74.1)	<0.0001
Married	63	175339	24.0 (17.8–30.2)	82	248142	32.9 (25.9–39.8)	
Cigarette smoking							
Never/former	193	537816	73.7 (67.7–79.6)	181	508705	67.4 (60.3–74.4)	<0.0001
Current	60	192331	26.3 (20.4–32.3)	72	246537	32.6 (25.6–39.7)	
Alcohol drinking							
Never/former	165	470411	64.4 (57.1–71.8)	168	448511	59.4 (51.6–67.2)	<0.0001
Current	88	259736	35.6 (28.2–42.9)	85	306732	40.6 (32.8–48.4)	
Occupation							
None	135	360160	49.3 (42.3–56.4)	161	442114	58.5 (50.8–66.2)	<0.0001
Blue collar	55	152765	20.9 (15.0–26.9)	44	115394	15.3 (10.2–20.4)	
White & pink collar	63	217222	29.8 (23.2–36.3)	48	197735	26.2 (19.0–33.4)	
Obesity							
Normal	168	490826	67.2 (60.9–73.5)	175	503318	66.6 (59.8–73.5)	0.6810
Obese	85	239321	32.8 (26.5–39.1)	78	251925	33.4 (26.5–40.2)	
Body mass index (kg/m2)							
<18.5	8	29232	4.0 (0.9–7.2)	16	57237	7.6 (3.3–11.9)	<0.0001
18.5–24.9	142	403287	55.9 (48.9–62.8)	165	456406	60.4 (53.1–67.8)	
<24.9	101	289543	40.1 (33.5–46.7)	72	241599	32.0 (24.9–39.0)	
Diabetes mellitus							
Normal	116	322405	51.0 (43.5–58.6)	140	432916	63.6 (56.4–70.9)	<0.0001
Impaired fasting glucose	64	202124	32.0 (24.7–39.3)	47	139713	20.5 (14.4–26.6)	
Diabetes mellitus	38	107251	17.0 (10.7–23.3)	37	107839	15.8 (10.4–21.3)	

Non-RA and RA group PS matched for sex and age.

**Table 2:** Matched conditional logistic regression of prevalence of RA.

	OR^A^	95% CI	*p* value
Prevalence of respiratory allergic diseases			
No respiratory allergic diseases	REF		
Respiratory allergic diseases	1.52	(1.31–1.75)	<0.001
Sex			
Male	REF		
Female	1.42	(1.11–1.83)	0.006
Age (years)			
<41	REF		
41–59	1.17	(0.81–1.70)	0.410
59<	0.95	(0.67–1.35)	0.788
Household income (1,000 KRW)			
<200	REF		
200–450	0.51	(0.47–0.56)	<0.001
450<	1.23	(1.09–1.38)	<0.001
Cigarette smoking			
Never/former	REF		
Current	1.51	(1.24–1.85)	<0.001
Alcohol drinking			
Never/former	REF		
Current	1.44	(1.27–1.63)	<0.001
Occupation			
None	REF		
Blue collar	0.56	(0.49–0.63)	<0.001
White & pink collar	0.69	(0.59–0.80)	<0.001
Obesity			
Normal	REF		
Obese	1.30	(1.18–1.43)	<0.001
Body mass index (kg/m^2^)			
<18.5	REF		
18.5–24.9	0.83	(0.63–1.10)	0.190
<24.9	0.65	(0.49–0.86)	0.002
Diabetes mellitus			
Normal	REF		
Impaired fasting glucose	0.53	(0.48–0.59)	<0.001
Diabetes mellitus	0.79	(0.70–0.88)	<0.001

Adjusted for sex, age, household income, cigarette smoking, alcohol drinking, occupation, obesity, BMI, and diabetes mellitus; OR^A^, adjusted OR; CI, confidence interval.

**Table 3:** Stratified PS matched conditional logistic regression of prevalence of RA.

	Number of events	OR^C^	95% CI	*p* value	OR^A^	95% CI	*p* value
No respiratory allergic disease		REF			REF		
Allergic rhinitis	20	1.37	(0.98–1.90)	0.064	1.39	(1.16–1.67)	<0.001
Asthma	11	2.81	(2.60–3.05)	<0.001	3.12	(2.77–3.51)	<0.001
Allergic rhinitis without asthma	18	1.07	(0.75–1.51)	0.720	1.03	(0.83–1.28)	0.773
Asthma without allergic rhinitis	9	1.87	(1.72–2.03)	<0.001	1.92	(1.72–2.14)	<0.001

Adjusted for sex, age, household income, cigarette smoking, alcohol drinking, occupation, obesity, BMI, and diabetes mellitus; OR^C^, crude OR; OR^A^, adjusted OR; CI, confidence interval.

## Abbreviations

AHR: Adjusted hazards ratio
BMI: Body mass index
CI: Confidence interval
DTH: Delayed-type hypersensitivity
IgE: Immunoglobulin E
IFN-gamma: Interferon-gamma
IL-2: Interleukin-2
IL-4: Interleukin-4
IL-5: Interleukin-5
IL-10: Interleukin-10
IL-13: Interleukin-13
KCDC: Korea Centers for Disease Control and Prevention
KNHANES: Korean National Health and Nutritional Examination Survey
MoHW: Ministry of Health and Welfare
NKG2D: Natural killer group 2D
OR: Odds ratio
OR^C^: Crude OR
OR^A^: Adjusted OR
RA: Rheumatoid arthritis
RR: Risk ratio
Th1: T-helper 1
Th2: T-helper 2
TNF-alpha: Tumor necrosis factor alpha
T-reg: T-regulatory
WC: Waist circumference.

## Additional Points

*Key Messages*. (i) Prevalence of respiratory allergic diseases, especially asthma, has an increased risk of RA prevalence. (ii) Th1 and Th2 diseases may coexist, where one may stimulate the counterpart's development. (iii) Prevalence of respiratory allergic diseases could be used as prevalence of RA's predicting factor.
